# Actionable gene-based classification toward precision medicine in gastric cancer

**DOI:** 10.1186/s13073-017-0484-3

**Published:** 2017-10-31

**Authors:** Hiroshi Ichikawa, Masayuki Nagahashi, Yoshifumi Shimada, Takaaki Hanyu, Takashi Ishikawa, Hitoshi Kameyama, Takashi Kobayashi, Jun Sakata, Hiroshi Yabusaki, Satoru Nakagawa, Nobuaki Sato, Yuki Hirata, Yuko Kitagawa, Toshiyuki Tanahashi, Kazuhiro Yoshida, Ryota Nakanishi, Eiji Oki, Dana Vuzman, Stephen Lyle, Kazuaki Takabe, Yiwei Ling, Shujiro Okuda, Kohei Akazawa, Toshifumi Wakai

**Affiliations:** 1 Division of Digestive and General Surgery, Niigata University Graduate School of Medical and Dental Sciences, 1-757 Asahimachi-dori, Chuo-ku, Niigata City, Niigata 951-8510 Japan; 2 Department of Gastroenterological Surgery, Niigata Cancer Center Hospital, 2-15-3 Kawagishi-cho, Chuo-ku, Niigata City, Niigata 951-8566 Japan; 3 Department of Breast Oncology, Niigata Cancer Center Hospital, 2-15-3 Kawagishi-cho, Chuo-ku, Niigata City, Niigata 951-8566 Japan; 4 Department of Surgery, Keio University School of Medicine, 35 Shinano-machi, Shinjyuku-ku, Tokyo, 160-8582 Japan; 5 Department of Surgical Oncology, Gifu University Graduate School of Medicine, 1-1 Yanagido, Gifu, 501-1194 Japan; 6 Department of Surgery and Science, Graduate School of Medical Sciences, Kyushu University, 3-1-1 Maidashi, Higashi-ku, Fukuoka, 812-8582 Japan; 7 Division of Genetics, Department of Medicine, Brigham and Women’s Hospital and Harvard Medical School, Boston, Massachusetts 02115 USA; 8 Broad Institute of Harvard and MIT, Cambridge, Massachusetts 02142 USA; 9 Molecular, Cell & Cancer Biology, University of Massachusetts Medical School, 55 Lake Avenue North, Worcester, Massachusetts 01655 USA; 10 Breast Surgery, Roswell Park Cancer Institute, Elm & Carlton Streets, Buffalo, New York 14263 USA; 11 Department of Surgery, University at Buffalo the State University of New York, 100 High Street, Buffalo, New York 14203 USA; 12 Division of Bioinformatics, Niigata University Graduate School of Medical and Dental Sciences, 1-757 Asahimachi-dori, Chuo-ku, Niigata City, Niigata 951-8510 Japan; 13 Department of Medical Informatics, Niigata University Medical and Dental Hospital, 1-757 Asahimachi-dori, Chuo-ku, Niigata City, Niigata 951-8510 Japan

**Keywords:** Gastric cancer, Next-generation sequencing, Gene panel, Precision medicine, Actionable gene

## Abstract

**Background:**

Intertumoral heterogeneity represents a significant hurdle to identifying optimized targeted therapies in gastric cancer (GC). To realize precision medicine for GC patients, an actionable gene alteration-based molecular classification that directly associates GCs with targeted therapies is needed.

**Methods:**

A total of 207 Japanese patients with GC were included in this study. Formalin-fixed, paraffin-embedded (FFPE) tumor tissues were obtained from surgical or biopsy specimens and were subjected to DNA extraction. We generated comprehensive genomic profiling data using a 435-gene panel including 69 actionable genes paired with US Food and Drug Administration-approved targeted therapies, and the evaluation of Epstein-Barr virus (EBV) infection and microsatellite instability (MSI) status.

**Results:**

Comprehensive genomic sequencing detected at least one alteration of 435 cancer-related genes in 194 GCs (93.7%) and of 69 actionable genes in 141 GCs (68.1%). We classified the 207 GCs into four The Cancer Genome Atlas (TCGA) subtypes using the genomic profiling data; EBV (*N* = 9), MSI (*N* = 17), chromosomal instability (*N* = 119), and genomically stable subtype (*N* = 62). Actionable gene alterations were not specific and were widely observed throughout all TCGA subtypes. To discover a novel classification which more precisely selects candidates for targeted therapies, 207 GCs were classified using hypermutated phenotype and the mutation profile of 69 actionable genes. We identified a hypermutated group (*N* = 32), while the others (*N* = 175) were sub-divided into six clusters including five with actionable gene alterations: *ERBB2* (*N* = 25), *CDKN2A*, and *CDKN2B* (*N* = 10), *KRAS* (*N* = 10), *BRCA2* (*N* = 9), and *ATM* cluster (*N* = 12). The clinical utility of this classification was demonstrated by a case of unresectable GC with a remarkable response to anti-HER2 therapy in the *ERBB2* cluster.

**Conclusions:**

This actionable gene-based classification creates a framework for further studies for realizing precision medicine in GC.

**Electronic supplementary material:**

The online version of this article (doi:10.1186/s13073-017-0484-3) contains supplementary material, which is available to authorized users.

## Background

Gastric cancer (GC) is the third leading cause of cancer death worldwide, with the highest incidence in Eastern Asia [1]. Despite the recent advances in cytotoxic chemotherapy, unresectable or recurrent GC remains notorious for the dismal prognosis of patients, with median survival ranging from 13.0 to 16.6 months [2, 3]. A number of clinical trials of targeted therapies have been conducted to improve the outcome of unresectable or recurrent GC [4]. To date, only two drugs have demonstrated significant efficacy: trastuzumab, an anti-HER2 antibody [5], and ramucirumab, an anti-VEGF receptor 2 antibody [6, 7]. The other targeted therapies failed to show any survival benefit in phase III clinical trials, deemed most likely due to the heterogeneous nature of GC and the lack of appropriate molecular biomarkers. Therefore, a better understanding of the molecular profiles of GC and optimum patient selection based on the molecular alterations is necessary to facilitate targeted therapies for GC.

Large-scale comprehensive molecular profiling using next-generation sequencing (NGS) technologies has identified the molecular landscape of a number of cancers, including GC [8–13]. The Cancer Genome Atlas (TCGA) cohort classified heterogeneous GC into four molecular subtypes: Epstein-Barr virus positive tumors (EBV), tumors with microsatellite instability (MSI), tumors with chromosomal instability (CIN), and genomically stable tumors (GS) based on various technologies, including whole-exome sequencing (WES) [10]. On the other hand, the Asian Cancer Research Group (ACRG), using array-based gene expression profiling, identified four distinct molecular subtypes that were associated with patient survival and recurrence patterns after surgery: MSI, microsatellite stable (MSS) with epithelial-to-mesenchymal transition (EMT) signature, MSS with TP53 activation, and MSS without TP53 activation [12]. These classifications have enabled us to improve our understanding of the molecular profiles and heterogeneity of GCs. However, neither of these classifications were designed to optimize patient selection for targeted therapies. Furthermore, comprehensive analyses such as WES and array-based profiling, as performed in the TCGA and ACRG projects, may not be feasible for clinical use due to their higher cost and generation of excessive information about molecular alterations of unknown biological and clinical significance. For clinical genetic testing, which is essential for precision medicine in cancer therapy, panel-based targeted sequencing using NGS technologies is advantageous because of precise target enrichment, enhanced depth of coverage, and reduced cost [14]. Thus, a clinically useful molecular classification based on targeted gene panel sequencing should help to realize precision medicine for GC.

We previously reported the clinical utility of genomic sequencing with a 415-gene panel in colorectal cancer [15]. We expanded the target genes to 435, including 69 genes that are associated with the US Food and Drug Administration (FDA)-approved targeted therapies (actionable genes), and added the evaluation of EBV infection and MSI status using NGS technologies. Here, we generated genomic profiles of 207 Japanese GCs using the 435-gene panel. Further hierarchical clustering based on actionable gene alterations successfully classified GCs into clusters directly associated with promising targeted therapies.

## Methods

### Patient inclusion criteria

A total of 207 patients histologically diagnosed with GC between 2009 and 2015 at Niigata University Medical and Dental Hospital, Niigata Cancer Center Hospital, Gifu University Hospital, Kyushu University Hospital, and Keio University Hospital were enrolled. The clinicopathological data for the individual patients are summarized in Additional file 1: Table S1. The study included 146 males and 61 females, with a median age of 66 years (range, 27–87 years). All patients, except for one patient with liver metastasis, underwent surgical resection with curative intent. Tumors were histologically staged according to the 7^th^ edition of the International Union against Cancer tumor-node-metastasis (TNM) classification system [16]. Collection and use of all specimens in this study were approved by the Institutional Review Boards of Niigata University, Niigata Cancer Center Hospital, Gifu University, Kyushu University, and Keio University. Informed consent was obtained from all subjects.

### Sequencing library preparation

Archival tissue in the form of formalin-fixed, paraffin-embedded (FFPE) tumor obtained during routine biopsy (*N* = 5) and/or surgical resection (*N* = 202) was used for analysis. An independent pathologist evaluated tumor content on hematoxylin and eosin-stained slides for each study sample to ensure > 20% tumor content was present. Where applicable, unstained slides were macro-dissected to enrich for tumor content, and DNA was extracted using a BioStic FFPE Tissue DNA Isolation Kit (MO BIO Laboratories, Carlsbad, CA, USA). All sample preparation, genomic sequencing, and analytics were performed in a CLIA/CAP-accredited laboratory (KEW, Cambridge, MA, USA).

### Panel-based genomic sequencing

DNA (50–150 ng) fragment libraries were prepared and enriched for the 435-gene panel with CANCERPLEX (KEW). CANCERPLEX is a clinically validated 435 gene panel enriched for coding regions and selected introns of genes with known association in cancer (Additional file 2: Table S2). Sequencing was performed on the Illumina MiSeq and NextSeq platforms (Illumina, San Diego, CA, USA) with average 500× sequencing depth. The CANCERPLEX DNA data sequencing pipeline, GENEPIPER v5.1, implements a series of public and proprietary algorithms designed to analyze DNA sequencing information. The analysis starts with demultiplexing by bcl2fastq v2.17.1.14 from Illumina, in order to obtain one FASTQ file per input sample. The resulting FASTQ files are realigned to the human reference sequence [University of California Santa Cruz (UCSC) hg19/GRCh37 assembly] using the Burroughs-Wheeler Alignment tool (BWA-MEM) in single-end mode [17, 18]. Duplicated reads resulting from PCR over-amplification or optical duplication are flagged and discarded by the MarkDuplicates module of Picard tools version 2.5 (http://broadinstitute.github.io/picard/).

Somatic and potential germline single nucleotide variants (SNVs) and short insertions and deletions (indels) are called from the cancer tissues using a proprietary combination of somatic and germline variant callers. The models used for this purpose take into account population genetics parameters as well as technical artifacts observed in the panel of normal samples and also statistics from databases such as the Genome Aggregation Database (gnomAD), 1000 Genomes, dbSNP, and COSMIC. All SNV and indel calling, including somatic and pathogenic potentially germline variants, was performed only in genomic regions intended to be captured by the assay (region of interest (ROI)).

Somatic copy number alterations (SCNAs) are called by calculating the number of mapped reads and then performing normalization using the panel of normal samples (49 samples), GC-content, repeat sequence, probe density, and other parameters. Copy number variants are called for exons as well as globally, using a backbone of probes throughout the genome. We segment regions using a Fused-Lasso method and export the results to a VCF file. The threshold for gains was > 2.5-fold and for loss < 0.75-fold. Fused genes (structural variants (SVs)) were detected only if at least one end mapped to any of the following genes: AKT3, ALK, BRAF, EGFR, ETV1, ETV4, ETV5, ETV6, FGFR2, FGFR3, MET, NOTCH1, NOTCH2, NRG1, PDGFRA, RAF1, RET, ROS1, TMPRSS2. Variants were filtered or flagged according to technical quality (e.g., coverage, allelic fraction, number of supporting reads), presence in previously characterized normal samples, or presence/absence in the following databases: dbSNP, ExAC, COSMIC, ClinVar, KEW. SNVs and indels in VCF format were annotated using ClinEff 1.0e and hg19 database (UCSC reference with RefSeq identifiers) and the output was adapted according to HGVS recommendations.

The tumor mutation burden (TMB), defined as the rate of peptide-changing SNVs per megabase, was determined for all tumors. To estimate the TMB, after standard filtering had been applied, only SNVs with a mutation allelic fraction of at least 10% and with high or moderate putative impact were retained. Tumors were tested for the presence of microsatellite instability (MSI) based on an extended loci panel. In addition to the Bethesda panel, a collection of 950 regions consisting of tandem repeats of one, two, or three nucleotides of minimum length of 10 bases. The number of indels within the ROI was calculated and tumors were classified as MSI-high (MSI-H) or microsatellite stable (MSS).

Tumors were also analyzed for the presence of HPV-16, HPV-18, and EBV (HHV-4) viral sequences. The reference genomes used were GI:310698439, GI:9626069, and NC_007605 for HPV-16, HPV-18, and EBV, respectively. The percentage of total number of reads mapped to the viral genomes was calculated and samples were designated as positive based on empirical cutoffs of 0.02, 0.01, and 0.0005% of reads that were mapped to HPV-16, HPV-18, and EBV genomes, respectively.

The specificity and sensitivity of all variant and biomarker detection was validated as previously described [19]. All non-synonymous genomic changes with low population frequency and allele fraction above 10% underwent a semi-automated curation and annotation process in GENEKEEPER, a proprietary curation tool and database used to determine pathogenicity and clinical relevance of alterations. We use the catalogue of FDA-approved drugs, the NCCN treatment guidelines, multiple mutation databases, and current scientific literature to determine if the variant protein is a target of a FDA-approved drug, a target of a drug in clinical development, or confers resistance to known treatments. Clinical trials are identified using ClinicalTrial.gov and other tools (e.g., Thompson Reuter Cortellis and Pharma Intelligence Trialtrove). Each variant is classified according the Association of Molecular Pathology (AMP) guidelines for somatic cancer variants [19]. Any variants classified as benign or likely benign were excluded from this study. To align mutations with their protein domains, genomic data were analyzed in Mutation Mapper (http://www.cbioportal.org/) for *ERBB2* in Japanese GC.

### Genomic profiles of 435 genes in TCGA GCs

Genomic data and clinicopathological characteristics from a total of 295 tumor samples of GC published in the TCGA database were downloaded from cBioPortal (http://www.cbioportal.org/) for inclusion in this study. To compare the clinicopathological characteristics between Japanese and TCGA GC, two-tailed Fisher’s exact test and Mann–Whitney U-test were applied for categorical and continuous variables, respectively. We down-sampled whole exome sequencing data to the 435 genes within the CANCERPLEX panel, including mutations (*N* = 289) and putative copy-number alterations from GISTIC (*N* = 293) for tumors that had sequencing data. The Oncoprint analysis tool (http://www.cbioportal.org/) was used to plot mutational data among different tumors. We evaluated mutation burden using the mutation data limited to 435 panel genes from TCGA GC samples (*N* = 289). STAD mutation data for the TCGA GCs were downloaded from the Broad GDAC Firehose website (https://gdac.broadinstitute.org/). Similar to the 435-gene panel bioinformatics pipeline, silent mutations that were not protein-altering were removed from the dataset. To compare mutation burden of the 435 gene panel to TCGA WES data on the same samples, the dataset of SNPs was down-sampled to the 435 genes in the panel, and the mutation rate determined in the panel was calculated as mutations/Mb. Correlations between the mutation rate of the 435 genes and of TCGA WES data were evaluated using the Spearman correlation coefficient.

### Gene clustering analysis

Mutation data from non-hypermutated Japanese GCs (*N* = 175) were extracted and clustered by mutated gene patterns using a method described previously [15]. Two different sets of genes—all 435 panel genes and 69 actionable genes—were used for clustering. The number of mutated genes in common related to donors i and j is presented as an element cij of an *N* × N matrix, where N is the number of non-hypermutated donors. In order to normalize the elements of this N dimension symmetric matrix into values ranging from 0 to 1, the original element was replaced by 1/(cij + 1), which indicates the level of similarity between donors i and j. Because of this normalization, donors with more mutated-genes in common would more likely come from a relatively close group. Consequently, a matrix with the normalized values between all donors was created. Hierarchical clustering of the matrix was performed for classifying donor groups with different mutated gene patterns by Euclidean distance and Ward’s clustering. This clustering was performed using R (https://www.r-project.org/). Clustering stability was evaluated by R package clValid for statistical and biological validation of clustering results, and the most stable clusters were determined [20].

### Statistical analysis of clinicopathological characteristics

To estimate associations between co-mutated gene patterns and clinicopathological characteristics, two-tailed Fisher’s exact test was applied to categorical variables by comparing the distribution in a cluster group to that of all the donors in the other groups. Mann–Whitney U-test was used for continuous variables. Note that in the case of statistical signature for hypermutated donors, two-tailed Fisher’s exact test was conducted against non-hypermutated donors as a reference set. All statistical tests were performed using the R package statmod (https://cran.r-project.org/web/packages/statmod/index.html), and differences with *P* < 0.05 are regarded as statistically significant.

## Results

### Overall genomic alterations detected using the 435-gene panel in Japanese GCs

Comprehensive genomic sequencing using the 435-gene panel detected at least one alteration of a cancer-related gene in 194 out of 207 patients (93.7%). Regarding 69 actionable genes which associated with FDA-approved targeted therapies, at least one alteration was detected in 141 patients (68.1%). Among 435 genes, mutations (single nucleotide substitutions (SNPs) and/or indels), somatic copy number alterations (SCNAs), and both alterations were found in 173, 31, and 30 genes, respectively. In 207 Japanese GC patients, the most frequently mutated gene was *TP53* (53.1%), followed by *ARID1A* (15.9%) and *CDH1* (14.0%). *ERBB2* amplification (12.1%) was the most frequently observed SCNA, followed by *CCNE1* (7.2%) and *KRAS* (5.8%) amplification (Table 1).

**Table 1:** Frequent gene alterations in 207 Japanese gastric cancers

Number	Mutation gene	Frequency	SCNA gene	Alteration	Frequency
1	*TP53*	53.1%	***ERBB2***	AMP	12.1%
2	*ARID1A*	15.9%	*CCNE1*	AMP	6.8%
3	*CDH1*	14.0%	***KRAS***	AMP	5.8%
4	***BRCA2***	10.6%	*ZNF217*	AMP	5.8%
5	*ARID1B*	10.1%	***CDKN2A***	DEL	5.3%
6	***ATM***	9.7%	***CDKN2B***	DEL	5.3%
7	***PIK3CA***	8.7%	*GATA4*	AMP	4.3%
8	*APC*	8.2%	*MYC*	AMP	2.4%
9	*ACVR2A*	7.2%	***CCND3***	AMP	1.9%
10	*CHD2*	6.3%	***CD274***	AMP	1.9%
11	*KMT2D*	6.3%	***CDK6***	AMP	1.9%
12	***RNF43***	5.8%	***EGFR***	AMP	1.9%
13	*EPHA2*	5.8%	***FGFR2***	AMP	1.9%
14	*TGFBR2*	5.3%	***JAK2***	AMP	1.9%
15	***FLCN***	4.3%	*GNAS*	AMP	1.9%
16	***PALB2***	4.3%	***CCND1***	AMP	1.4%
17	*PTPRT*	4.3%	***MET***	AMP	1.4%
18	*RAD50*	4.3%	*HSP90AB1*	AMP	1.4%
19	***BRCA1***	3.9%	*SMAD4*	DEL	1.4%
20	***STK11***	3.9%	*TEK*	DEL	1.4%

*AMP* amplification, *DEL* deletion, *SCNA* somatic copy number alteration

Bold gene symbols indicate genes from the 69 actionable genes for FDA-approved targeted therapies

### Molecular classification according to TCGA subtype in Japanese GCs

**Fig. 1:**
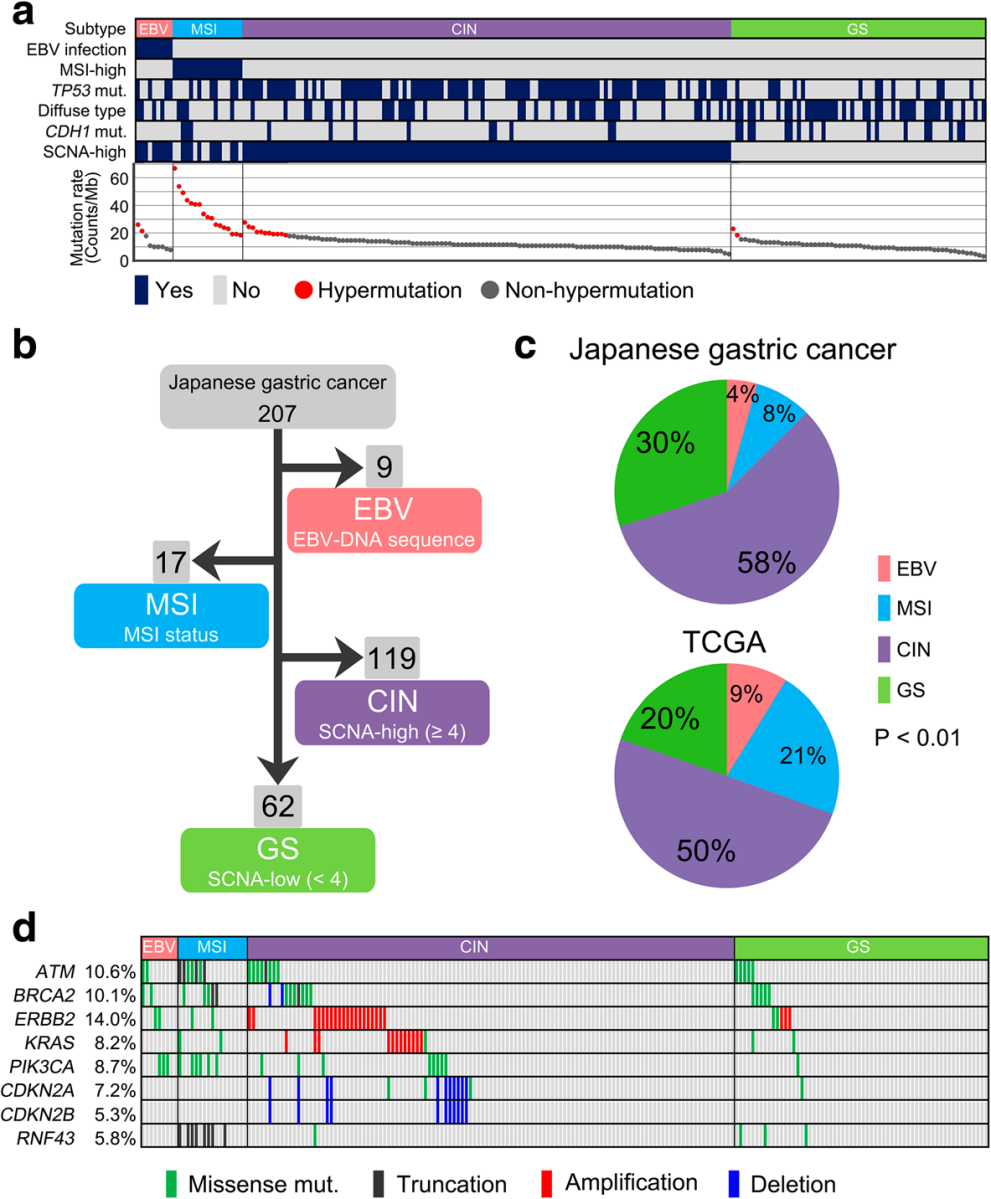
The Cancer Genome Atlas molecular subtypes in 207 Japanese gastric cancers (GCs). **a** The 207 Japanese GCs are classified into four molecular subtypes; Epstein-Barr virus-positive (*EBV*, *red*), tumor with microsatellite instability (*MSI*, *blue*), tumors with chromosomal instability (*CIN*, *purple*), and genomically stable tumors (*GS*, *green*). Color tiles indicate pathological and molecular characteristics of of GC. Tumors are ordered by mutation rate and the *red dots* indicate hypermutated tumors with a mutation rate of more than 18.5 counts/Mb, which was the lowest value in the MSI subtype. **b** Classification of 207 Japanese GCs into TCGA molecular subtypes. The cutoff value of somatic copy number alterations (SCNAs) is defined as four loci according to the status of TP53 mutation and histological type (Additional file 3: Figure S1). **c** Distribution of molecular subtypes in 207 Japanese (*upper*) and TCGA (*lower*) GCs. **d** Landscape of frequently observed actionable gene alterations (5% or more) in 207 Japanese GCs classified by TCGA subtype. Alteration color indicates the class of gene alterations

The 207 Japanese GCs were classified into four TCGA subtypes based on the genomic profiling data from the 435-gene panel (Fig. 1a). Nine tumors (4%) positive for EBV-DNA sequence were classified as EBV subtype. MSI-high (MSI-H) status was found in 17 tumors (8%), and they were classified as MSI subtype. Finally, the remaining 181 tumors were divided into CIN and GS subtypes using SCNA status. In the previous TCGA study, *TP53* mutations were frequently observed in the CIN subtype, and tumors with a diffuse type of Lauren classification were enriched in the GS subtype. To match this classification scheme, we defined SCNA-high tumors as those with four or more SCNA loci (Additional file 3: Figure S1). As a result, 119 SCNA-high tumors (58%) were categorized as CIN subtype, and the remaining 62 tumors (30%) with SCNA-low as GS subtype (Fig. 1b). We compared the distribution of the four subtypes between Japanese and TCGA GCs. Regarding regional differences between enrolled patients, the nationality of 75% of patients in the TCGA study is European or American (Additional file 3: Figure S2a); on the other hand, all patients included in our study were Japanese. However, the clinicopathological characteristics of each subtype of Japanese GC were roughly comparable to those of the TCGA GCs (Additional file 3: Figure S2b–f). The proportion of the MSI subtype was lower, and that of the GS subtype was higher, in Japanese GCs than in TCGA GCs (*P* < 0.01; Fig. 1c). Because genomic profiling of actionable genes may lead to strategies for optimal targeted therapies in GC, we examined actionable gene alterations that were observed at a frequency of 5% or more in Japanese GCs. Interestingly, these alterations were not specific to a TCGA subtype (Fig. 1d).

### Hypermutated tumors in Japanese GCs

**Fig. 2:**
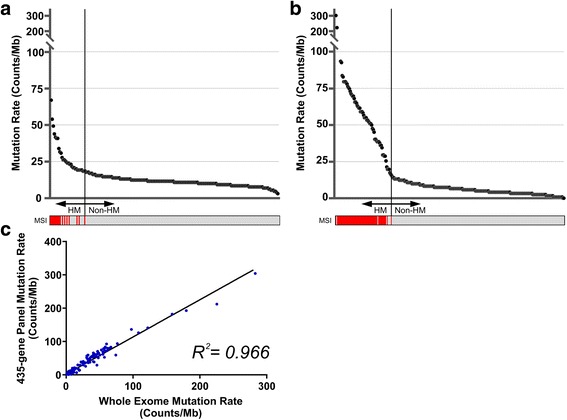
Mutation rates in Japanese and The Cancer Genome Atlas gastric cancers. **a** Mutation rate from Japanese GCs was determined by the number of non-synonymous SNVs in the 435-gene panel. *Red*, MSI-H; *gray*, MSS; *HM*, hypermutation. **b** WES data from TCGA GCs was down-sampled to the content of the 435-gene panel. *Red*, MSI-H; *gray*, MSS; *HM*, hypermutation. **c** Correlation between mutation rates determined using the 435-gene panel and WES data of TCGA GCs

Recently, hypermutated tumors have been considered to have a higher response to immunotherapy because of the development of neo-antigens [21, 22]. In the previous TCGA study of GC, hypermutated tumors were defined as MSI-H tumors [10]. The mutation rate of MSI-H tumors (31.5 counts/Mb, range 18.5–66.9) was significantly higher than that of MSS tumors (11.2 counts/Mb, range 3.1–27.7) in Japanese GCs (*P* < 0.01). We defined hypermutated tumors as those with a mutation rate of more than 18.5 counts/Mb, which was the lowest value in MSI-H tumors (Fig. 2). To validate the cutoff value of mutation rate, WES data of TCGA GCs was down-sampled to the 435 genes, and mutation rate calculated. A high correlation of mutation rate between WES and the 435-gene panel was demonstrated (correlation coefficient of 0.966). The lowest mutation rate of MSI-H tumors in TCGA GCs was 16.4 counts/Mb, and was nearly equivalent to that in Japanese GCs. As a result, we identified 32 tumors (15.5%) as hypermutated tumors, which not only included the MSI subtype but also some tumors from the EBV, CIN, and GS subtypes (Fig. 1a).

### Clustering based on gene alterations for targeted therapy in Japanese GCs

**Fig. 3:**
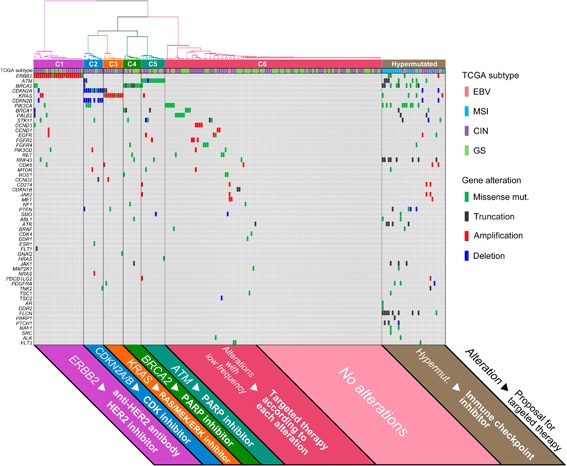
Clustering based on 69 actionable gene alterations. According to co-alteration patterns of the 69 actionable gene subset, 175 non-hypermutated tumors are divided into six clusters, and the gene alteration spectrum and TCGA molecular subtype of each tumor is demonstrated as color tiles. Alteration in color indicates the class of gene alteration. The 32 hypermutated tumors are shown on the *right* (*brown*). The specific gene alteration and proposal for targeted therapy are presented under each cluster. Fourteen genes which have no alterations in 207 tumors are not included in this figure

Actionable gene alterations and hypermutated phenotypes were widely observed throughout all TCGA subtypes in our analysis (Fig. 1a, d). Novel classifications, directly associated with these alterations, may better pinpoint candidates for targeted therapies than TCGA subtypes. Thus, our 207 tumors were classified using the hypermutated phenotype and alteration profile of the 69 actionable genes. Hypermutated tumors (*N* = 32) were identified, and the remaining non-hypermutated tumors (*N* = 175) were subdivided into six clusters by hierarchical clustering (Fig. 3). Firstly, tumors with *ERBB2* alterations were classified as cluster 1 (*N* = 25). Remaining tumors without *ERBB2* alterations were classified into two large clusters according to the enrichment of actionable gene alterations. The cluster with enrichment of the major alterations were subsequently divided into four clusters (clusters 2–5) based on intrinsic gene alterations in *CDKN2A/B* (*N* = 10), *KRAS* (*N* = 10), *BRCA2* (*N* = 9), or *ATM* (*N* = 12). Cluster 6 (*N* = 109) included tumors with minor alterations and those with no alterations in actionable genes. Using this clustering scheme, FDA-approved targeted therapies could be associated with a majority of tumors, including hypermutated tumors, those in clusters 1–5 and a part of cluster 6.

In addition, we performed hierarchical clustering based on the 435-gene alterations to assess the associations between overall genomic profile and clinicopathological features. Non-hypermutated tumors were classified into nine clusters (Additional file 3: Figure S3a). We identified the clusters which have specific clinicopathological features (Additional file 3: Figure S3b). Tumors in cluster 9 (*N* = 16), which are characterized by concomitant alterations of *TP53* and *ERBB2*, significantly associated with high counts of SCNA (*P* < 0.01) and the intestinal type of Lauren classification (*P* < 0.05) compared with tumors in all other clusters. On the other hand, tumors in cluster 4 with primary mutated gene CDH1 significantly associated with young age (*P* < 0.05), low counts of SCNA (*P* < 0.01), female gender (*P* < 0.01), and diffuse type of Lauren classification (*P* < 0.01). These findings suggest that genomic profiling generated by the 435-gene panel reflects the clinicopathological features in Japanese GCs.

### Details of gene alterations and a remarkable case in the ERBB2 cluster (cluster 1)

**Fig. 4:**
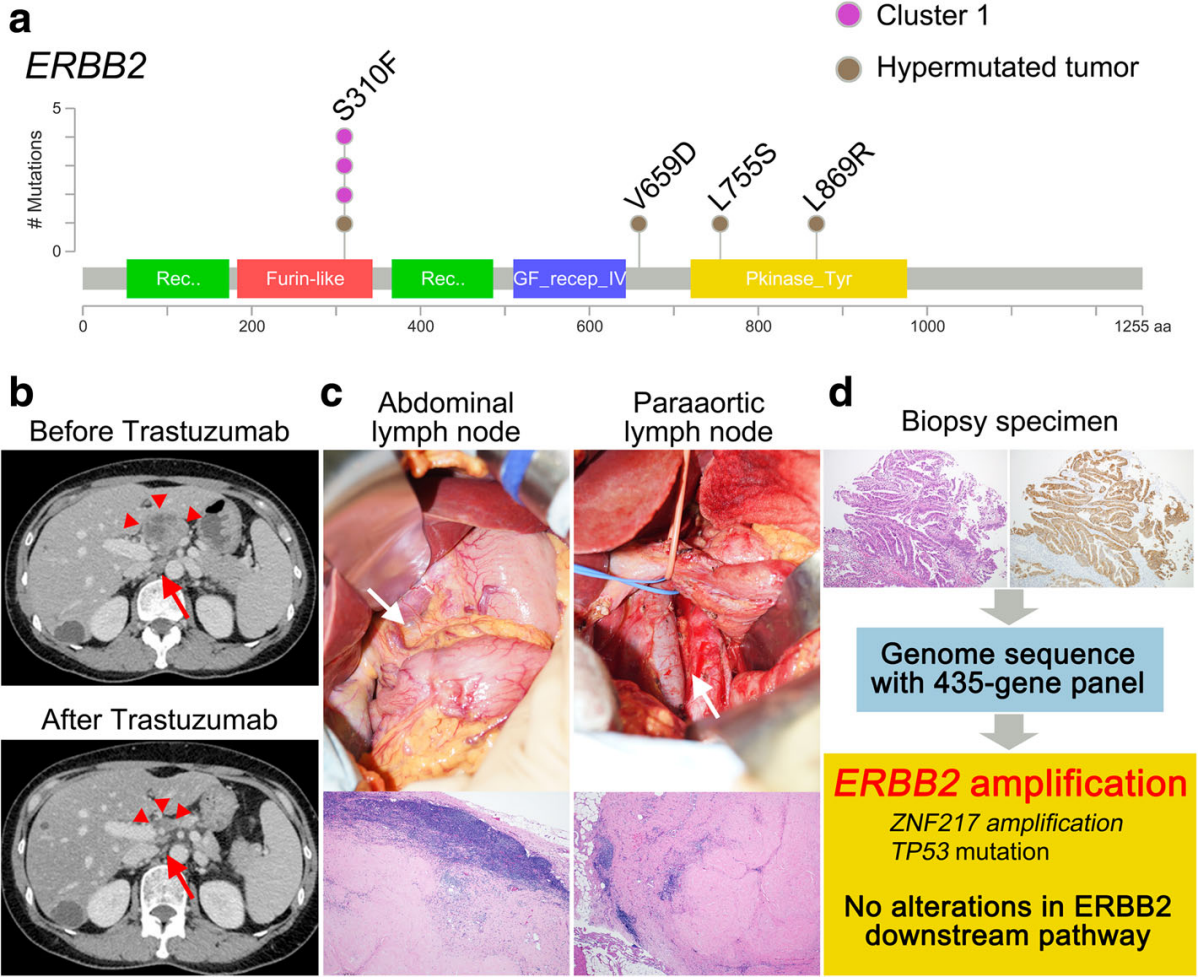
Details of *ERBB2* alterations and a case of remarkable response to anti-HER2 therapy in the ERBB2 cluster. **a** Mutations of ERBB2 identified in 207 Japanese GCs are aligned to the protein domain. Patient samples are further plotted by mutation status. **b** Abdominal enhanced CT before trastuzumab therapy (*upper panel*) demonstrated extensive metastases in abdominal (*arrow head*) and paraaortic (*arrow*) lymph nodes. All the metastatic lymph nodes were remarkably reduced in size after trastuzumab therapy (*lower panel*). **c** Abdominal lymph nodes were reduced in size, and paraaortic lymphadenectomy was performed (*upper panel*). Histological examination revealed no viable tumor cells in the abdominal and paraaortic lymph nodes (*lower panel*, hematoxylin and eosin, original magnification × 40). **d** Genomic sequencing in biopsy specimen obtained before treatment (*left panel*, hematoxylin and eosin; *right panel*, anti-HER2 antibody) identified *ERBB2* and *ZNF217* amplification and *TP53* mutation. However, concomitant alterations in the *ERBB2* downstream pathway were not observed in the tumor of this patient

Anti-HER2 therapy with trastuzumab is recommended as a first line therapy for HER2-overexpressing GC [5]. Tumors harboring *ERBB2* alterations were primarily assigned into cluster 1 in our study. *ERBB2* amplification, mutation, and both alterations were detected in 22, two, and one tumor, respectively. Details of *ERBB2* mutations in the Japanese GCs are shown in Fig. 4a. All the mutations detected in cluster 1 were S310F, which is well-known to activate HER2 signaling [23, 24]. Tumors with the S310F mutation have been reported to have good response to anti-HER2 therapy [25].

Within cluster 1, one patient demonstrated a remarkable response to anti-HER2 therapy (Fig. 4b–d). In brief, this patient was a 55-year old woman with unresectable GC because of extensive abdominal and paraaortic lymph node metastases. HER2 overexpression was confirmed by immunohistochemistry in the biopsy specimen, and trastuzumab combined with chemotherapy was administered. After seven courses of treatment, complete response according to RECIST ver. 1.1 was observed in abdominal and paraaortic lymph node metastases. The primary tumor of the stomach also decreased in size, although histological examination of the endoscopic biopsy specimen revealed residual tumor. A PET-CT scan showed abnormal uptake only in the primary tumor of the stomach. Therefore, we performed distal gastrectomy with regional and paraaortic lymphadenectomy, and achieved curative resection. Histological examination revealed no viable tumor cells in the abdominal and paraaortic lymph nodes. Genomic alterations in the biopsy specimen obtained before treatment were retrospectively examined using the 435-gene panel. Consistent with the immunohistochemistry, *ERBB2* amplification was detected. Other alterations detected were *ZNF217* amplification and *TP53* mutation. Concomitant alterations in the ERBB2 downstream pathway, which confer resistance to trastuzumab therapy, were not observed in the tumor of this patient.

## Discussion

GC is a highly heterogeneous disease with various histological phenotypes and molecular diversity. Intertumoral heterogeneity represents a significant hurdle to identifying optimized targeted therapies in GC. Stratification of patients based upon the distinctive genomic alterations in their tumors is needed to realize precision medicine in GC. To the best of our knowledge, the current study is the first to demonstrate the actionable gene-based molecular classification associated with FDA-approved targeted therapies using panel-based targeted sequencing. We generated comprehensive genomic profiling data for 207 Japanese GCs using a 435-gene panel including the evaluation of EBV infection and MSI status based on NGS technologies. We identified five clusters by hierarchical clustering according to alterations in 69 actionable genes in addition to hypermutated status, which are associated with FDA-approved targeted therapies. Our study highlighted the potential of panel-based targeted sequencing to provide reliable information for realizing precision medicine in GC.

Large-scale genome projects have attempted to identify distinct molecular subtypes to help define the heterogeneity of GCs; however, a clinically useful classification that is relevant to targeted therapies is yet to be found for GC. TCGA molecular subtypes are a reasonable classification which reflects the tumor biology and associates with clinicopathologic features of GC [10]. ACRG molecular subtypes, established using gene expression and genome-wide copy number microarray data, were associated with survival outcome and recurrence patterns in patients with GC after surgery [12]. However, it remains unclear whether these molecular subtypes can provide optimum targeted therapies in GC. In this study, 207 Japanese GCs were classified into TCGA molecular subtypes, and we showed that alterations in actionable genes are not specific to TCGA molecular subtype, but rather are widely distributed throughout the subtypes. Therefore, TCGA molecular subtype may not be directly useful for clinical applications to optimize targeted therapies. Novel classifications which more precisely select candidates for targeted therapies are necessary to realize precision medicine in GC.

We successfully classified 207 tumors into clusters directly associated with FDA-approved targeted therapies according to the alterations of 69 actionable genes and hypermutated phenotype. We identified the clusters with enrichment of *CDKN2A/B*, *KRAS*, *ATM*, or *BRCA2* alterations, in addition to *ERBB2* ones. *ATM* and *BRCA1/2* play an essential role in double-strand DNA break repair pathway, and the deficiency of this pathway caused by the alterations of these genes were associated with high sensitivity to the PARP inhibitors in solid tumors, including GC [26, 27]. A phase II clinical trial revealed the efficacy of olaparib as a second line therapy in recurrent or metastatic GC with negative expression of ATM protein by immunohistochemistry [28]. However, phase III clinical trial did not show the significant overall survival benefit of olaparib in this population (NCT01924533) [29]. Further clinical studies of PARP inhibitors for *BRCA1/2* or *ATM* mutated GCs are necessary to establish the appropriate biomarker and to reveal the benefit of this therapy. *KRAS* mutation and amplification lead to an activation of the RAF-MEK-ERK pathway, which is critical to pathogenesis and progression in many cancers [30]. KRAS itself is difficult to directly inhibit; therefore, inhibition of the downstream RAF-MEK-ERK pathway is considered a promising treatment for *KRAS*-mutated tumors [31]. A clinical benefit of the FDA-approved MEK inhibitor trametinib has been partially demonstrated in cancers with a *KRAS* mutation [32, 33]. *CDKN2A/B* protein products restrict cell-cycle progression by inhibiting CDK4/6 kinase activity, and aberrations in these genes lead to cancer progression by dysregulation of the cell cycle [34]. The association between *CDKN2A/B* deletion and the sensitivity to palbociclib, an FDA-approved CDK4/6 inhibitor, has been demonstrated in many cancers [35–38]. Therefore, it is worth investigating the clinical utility of the above-mentioned targeted therapies for GCs with corresponding gene alterations to establish precision medicine in GC.

Our hierarchical clustering based on actionable gene alterations identified the cluster with *ERBB2* alterations as one of the major clusters. Patients with tumors harboring *ERBB2* amplification (*N* = 22) are candidates for trastuzumab, an anti-HER2 monoclonal antibody, as a standard first line therapy for unresectable metastatic GC. Excitingly, a remarkable response to trastuzumab therapy was observed in one of the *ERBB2*-amplified tumors categorized into this cluster, and genomic sequencing identified no concomitant alterations in the ERBB2 downstream pathway. Alterations in the PI3K/mTOR and MAPK pathways and amplification of other receptor tyrosine kinases are proposed mechanisms of trastuzumab resistance [39–41]. Genomic profiling using the 435-gene panel enabled us to simultaneously examine the gene alterations of these pathways in single testing, and has the potential to provide reliable information to predict treatment response and to overcome resistance for targeted therapies [42, 43].

Recent advances on the genetics of GC are pointing toward an enrichment of the mutations involved in double-strand DNA break repair via homologous recombination (HR). Alexandrov et al. [44] demonstrated that 7–12% of GCs have deficiencies in this pathway using a large-scale mutational signatures analysis. Sahasrabudhe et al. [45] identified 11 of 362 GC cases with germline mutations in *PALB2*, *BRCA1*, or *RAD51C*, which regulate HR. In this study, we identified the high frequency of mutations in HR genes, including *BRCA1/2*, *ATM*, *PALB2*, and *RAD50* (Table 1). This results indicates that defective HR is a crucial genetic aberration in Japanese GCs, similar to recent studies. Ovarian and breast cancers with defective HR have high sensitivity to platinum agents as well as PARP inhibitors [46, 47]. Platinum-based chemotherapy is a mainstay of current treatment for advanced GC [2, 4]. Therefore, the assessment of defective HR using panel-based targeted sequencing might allow optimization of patient selection for platinum-based chemotherapy in GC.

Previous comprehensive studies characterized diffuse type GCs as tumors with a low frequency of mutation and SCNA, which were classified as GS subtype in the TCGA study [10] or MSS/EMT subtype in the ACRG study [12]. Promising therapeutic targets are hard to identify in these subtypes because of the genomic stability. Indeed, in our hierarchical clustering analysis, diffuse type and GS tumors were significantly enriched in 63 tumors with no actionable alterations which associated with FDA-approved targeted therapies: 52.4 and 46.0%, respectively (Additional file 3: Figure S4). Several studies using WES or WGS focusing on diffuse type GCs were conducted to understand the molecular background and to explore therapeutic targets [9, 48]. However, promising targeted therapies are not currently developed in diffuse type GCs. Further challenges exist in developing effective targeted therapies for these tumors with genomic stability.

Except for trastuzumab, targeted therapies proposed by our actionable gene-based classification are not currently approved for GC. Future studies are needed to determine the effectiveness of actionable gene-based classifications to improve patient outcomes. Clinical trials for targeted therapies, coupled with genomic profiling for optimum patient selection, are required to demonstrate clinical utility, including treatment outcome and cost-effectiveness. Nonetheless, we believe that our genomic profiling data and classification are informative for future studies to explore the rationale for precision medicine in GC because our study represents the most extensive large-scale targeted sequencing project in Japan where a high incidence of GC is observed.

## Conclusions

We generated genomic profiling data for 207 Japanese GCs using the 435-gene panel with NGS technology. Our actionable gene-based classification creates a framework for further studies aimed at realizing precision medicine in GC.

## Additional files

Additional file 1: Table S1. Clinicopathological characteristics of 207 Japanese gastric cancers. (XLSX 38 kb)

Additional file 2: Table S2. Detail of the 435-gene panel. (XLSX 16 kb)

Additional file 3: Figures S1-S4. **Figure S1.** Definition of somatic copy number alteration (SCNA) status. **Figure S2.** Clinicopathological characteristics of TCGA molecular subtype in Japanese and TCGA gastric cancers (GCs). **Figure S3.** Cluster of 435-gene co-mutation patterns. **Figure S4.** Distribution of Lauren classification and TCGA molecular subtypes by presence or absence of actionable gene alterations. (PDF 725 kb)

## Abbreviations

ACRG: The Asian Cancer Research Group
CIN: Chromosomal instability
EBV: Epstein-Barr virus
EMT: Epithelial-to-mesenchymal transition
FDA: US Food and Drug Administration
FFPE: Formalin-fixed, paraffin embedded
GC: Gastric cancer
GS: Genomically stable
HR: Homologous recombination
Indel: Insertion/deletion
MSI: Microsatellite instability
MSS: Microsatellite stable
NGS: Next-generation sequencing
ROI: Region of interest
SCNA: Somatic copy number alteration
SNP: Single nucleotide substitution
SNV: single nucleotide variant
TCGA: The Cancer Genome Atlas
TMB: Tumor mutation burden
TNM: Tumor-node-metastasis
WES: Whole-exome sequencing.
