# Supplementary material for: Actionable gene-based classification toward precision medicine in gastric cancer
Source: Genome Med. 2017 Oct 31;9:93. doi: 10.1186/s13073-017-0484-3 (PMC5664811; doi:10.1186/s13073-017-0484-3)
Supplement: Supplementary file 3 — Figure S1. Definition of somatic copy number alteration (SCNA) status. Figure S2. Clinicopathological characteristics of TCGA molecular subtype in Japanese and TCGA gastric cancers (GCs). Figure S3. Cluster of 435-gene co-mutation patterns. Figure S4. Distribution of Lauren classification and TCGA molecular subtypes by presence or absence of actionable gene alterations. (PDF 725 kb) [file 13073_2017_484_MOESM3_ESM.pdf]

Figure S1

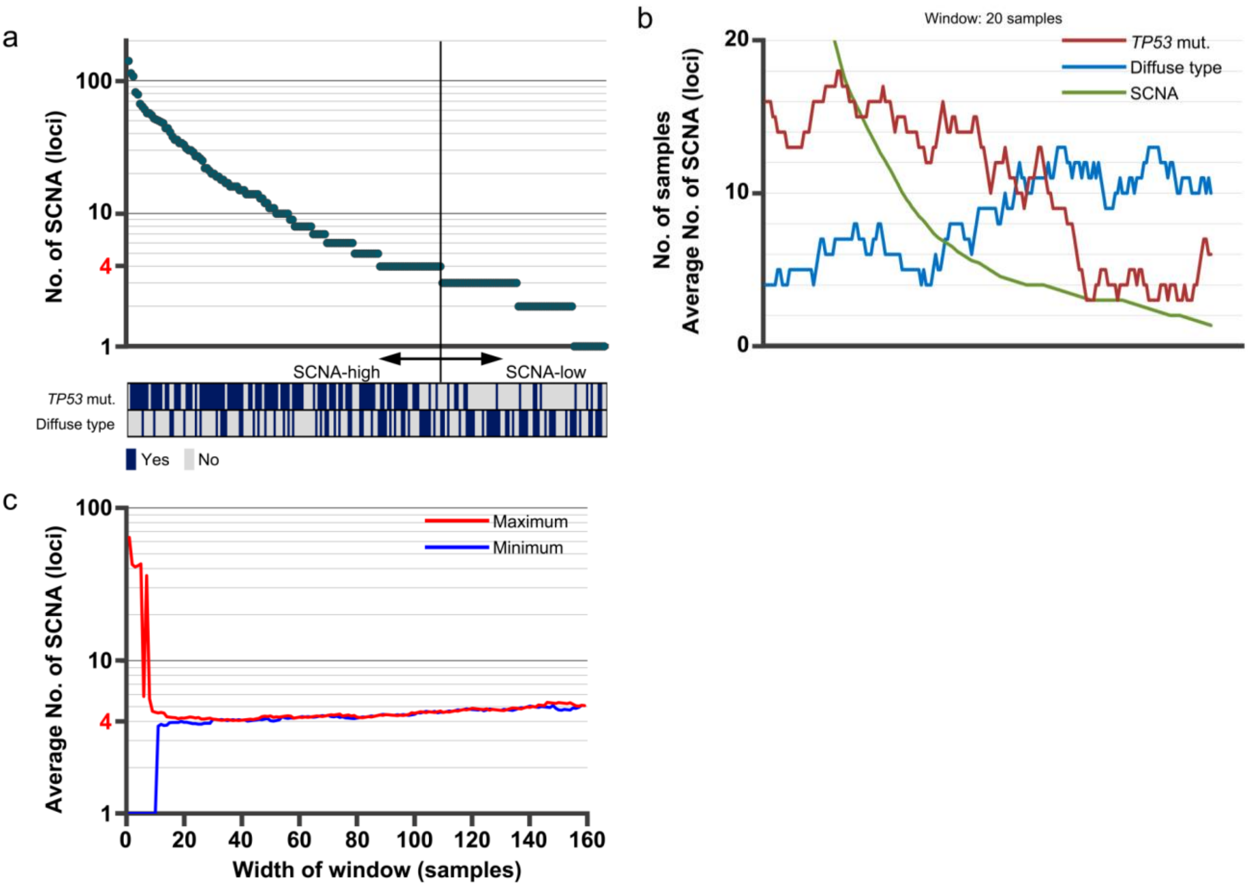

Figure S1: Definition of somatic copy number alteration (SCNA) status.

a. To define the SCNA-high and low tumor, *TP53* mutation and Lauren classification were evaluated. When ordering donors in descending order of SCNA frequency, the number of TP53 mutated and Lauren diffused type donors was summed in a given number of consecutive donors (2–160).

b. The values of these two indexes were calculated as a window search from the most frequently mutated donor to lower. Subsequently, the maximum and minimum number of SCNA at the intersection point between *TP53* and Lauren classification was decided.

c. The maximum and minimum SCNA values were plotted along to the ascending window sizes, saturating to around four of SNCA.

Figure S2

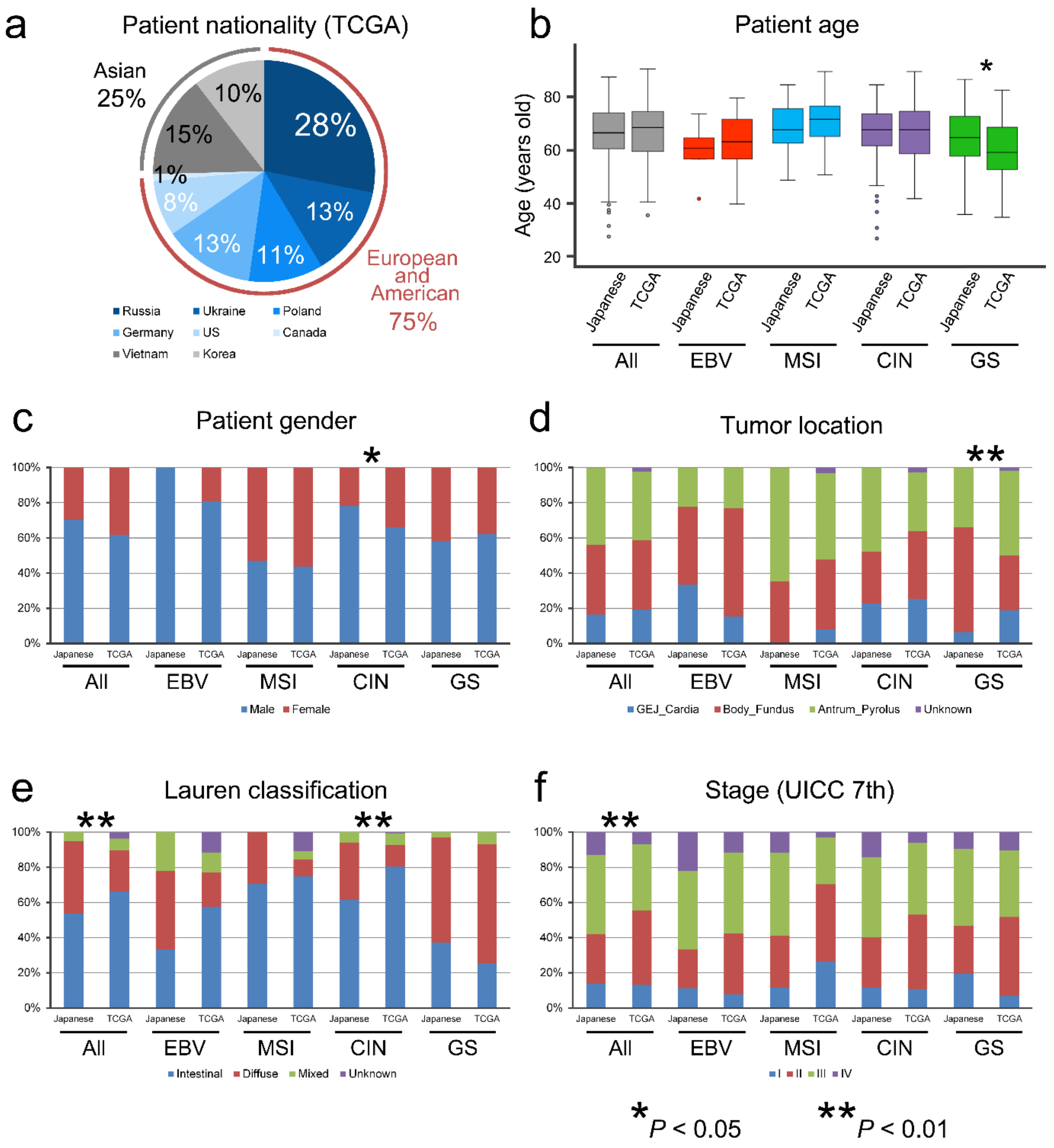

Figure S2: Clinicopathological characteristics of TCGA molecular subtype in Japanese and TCGA gastric cancer (GC).

Nationality of 75% of patients in TCGA GC was European and American countries (a). Patient age (b), gender (c), tumor location (d), Lauren classification (e) and Stage (f) were compared between Japanese and TCGA GC (**P* <0.05, ***P* <0.01).

Figure S3

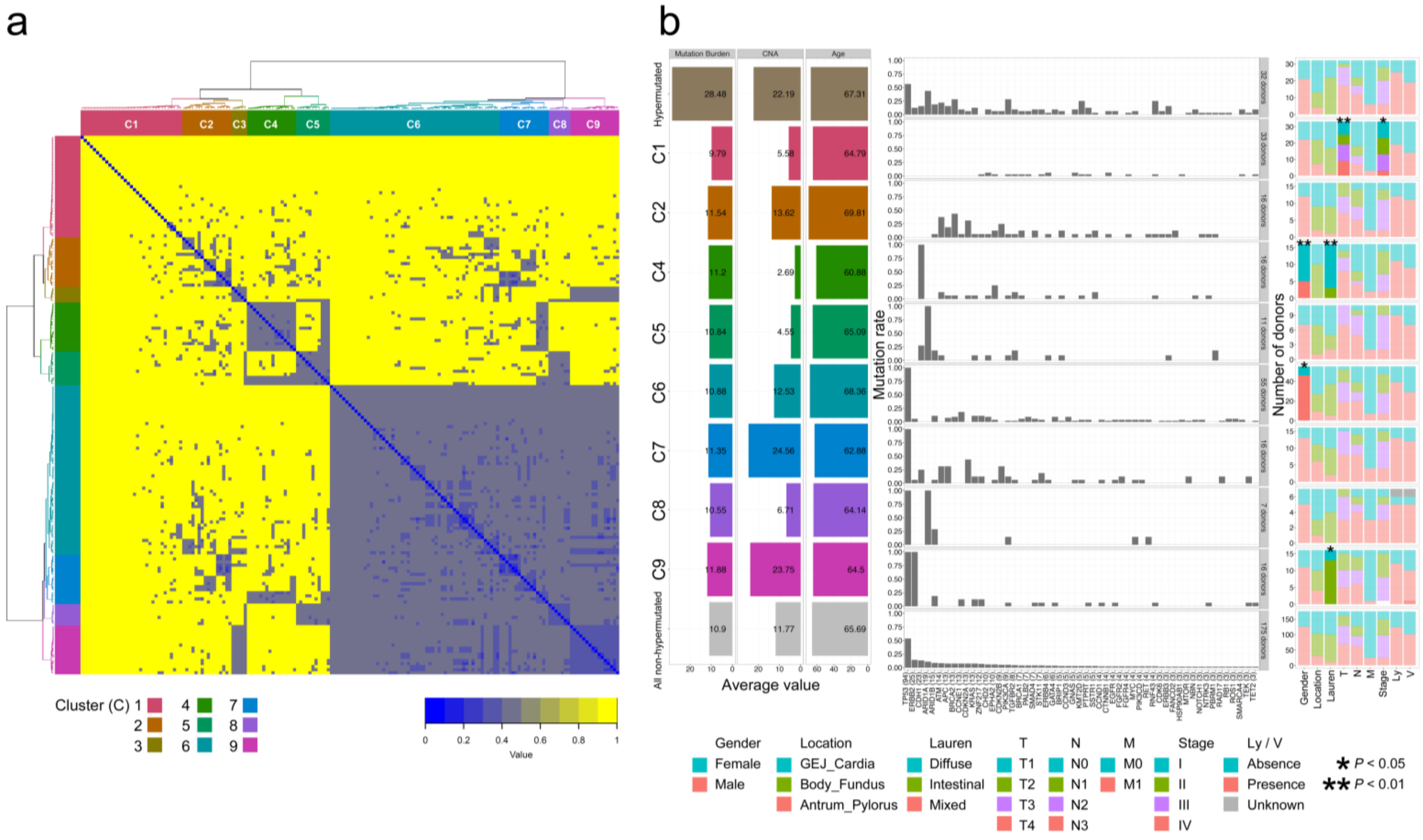

Figure S3: Cluster of 435-gene co-mutation patterns.

a. Cluster analysis was performed on non-hypermutated tumors (N = 175) by using Euclidean distance and Ward's clustering method (closest distance to common mutated genes are colored yellow to blue).

b. Co-mutated gene patterns of the 435-gene set with statistical analysis. Mutation rate in each group is shown as a bar graph in the middle panel. Group-based mean values for mutation burden, copy number alteration (CNA) and age are shown (left) with cluster colors and fraction for clinical information (right). Dark bars indicate significant difference (*P* <0.05, two-tailed Fisher's exact test) to the distribution of all other non-hypermutated donors and light bars are non-significant (**P* <0.05, ***P* <0.01).

Figure S4

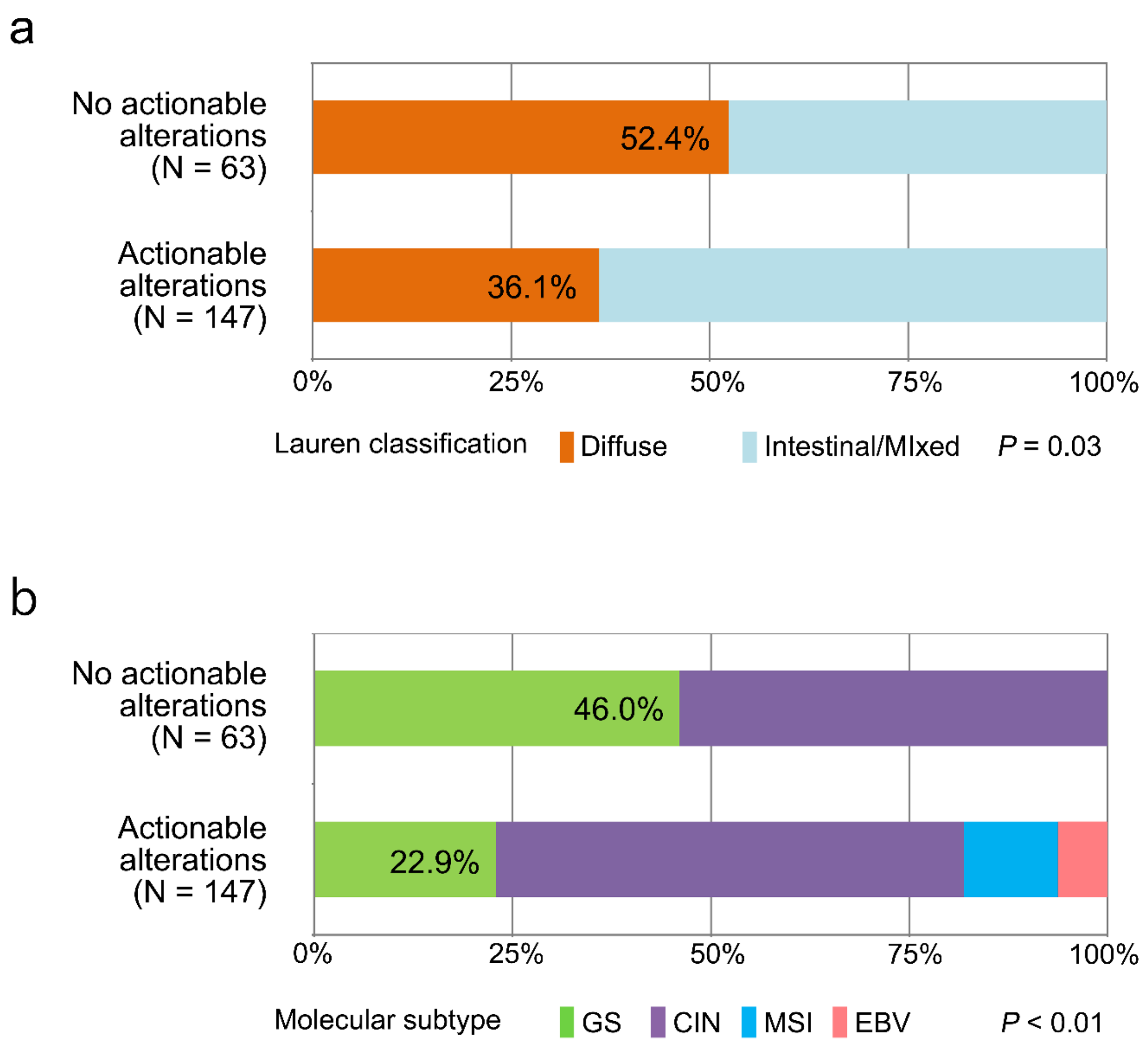

Figure S4: Distribution of Lauren classification (a) and TCGA molecular subtypes (b) by presence or absence of actionable gene alterations.
